# TRIB3, as a robust prognostic biomarker for HNSC, is associated with poor immune infiltration and cancer cell immune evasion

**DOI:** 10.3389/fimmu.2023.1290839

**Published:** 2024-01-03

**Authors:** Huadong Wu, Zhenzhen Fu, Hong Li, Feifei Fang, Bin He, Yujie Ye, Heyong Wu, Dong Xu, Haoran Zheng, Qiang Zhang

**Affiliations:** ^1^ Department of Oral and Maxillofacial Surgery, The First Affiliated Hospital, Jiangxi Medical college, Nanchang University, Nanchang, Jiangxi, China; ^2^ Department of Otolaryngology, The First Affiliated Hospital, Jiangxi Medical College, Nanchang University, Nanchang, Jiangxi, China

**Keywords:** tribbles pseudokinase 3 (TRIB3), head and neck squamous cell carcinoma (HNSC), prognostic biomarker, immune evasion, immune infiltration

## Abstract

**Objective:**

As a pseudokinase, Tribbles Pseudokinase 3 (TRIB3) is implicated in a wide array of biological processes, including cell signal transduction, metabolic regulation, stress responses, and immune regulation. While its significant role in the immune regulation of certain cancers is well-established, the specific functions and impact of TRIB3 in head and neck squamous cell carcinoma (HNSC) remain unclear.

**Methods:**

The data of RNA-sequence was acquired from the TCGA database to analyze the expression patterns of TRIB3 and elucidate its prognostic value in HNSC patients. Furthermore, the correlation between TRIB3 and tumor mutation burden, clinical data, immune checkpoint genes, and immune cell infiltration was explored. Moreover, the TRIB3 location in tumor tissues and subcellular structures was identified *via* Tisch in the HPA database, and the potential protein interaction molecules for TRIB3 were elucidated in the STRING database. The potential TRIB3 gene function was assessed using gene set enrichment analysis (GSEA), whereas the TRIB3 expression levels in clinical HNSC samples were verified by RT-qPCR and immunohistochemistry. the role of TRIB3 in enhancing the malignant behavior of HNSC cells was validated *in vitro* through a series of methods including RT-qPCR, CCK8 assay, wound healing assay, and transwell assay.

**Results:**

It was revealed that TRIB3 was significantly overexpressed in the nucleus and cytoplasm of HNSC. Furthermore, this overexpression markedly enhanced the migration ability of tumor cells. As an independent prognostic factor, TRIB3 was associated with advanced tumor T stage and was significantly involved with tumor mutation burden and immune cell infiltration in HNSC. Moreover, it was observed that TRIB3 was not a predicted factor for PD1/PDL1 and ATL4 inhibitor treatment; however, it was substantially correlated with various immune evasion-related genes in HNSC.

**Conclusion:**

TRIB3 could serve as a potential prognostic marker for HNSC and might be a key gene mediating HNSC immune evasion.

## 1 Introduction

Head and neck cancer is among the most prevalent cancers, with 90% of the cases corresponding to head and neck squamous cell carcinoma (HNSC) globally (1). In 2018, the global incidence of HNSC diagnoses exceeded 890,000, and the total number of reported deaths was around 450,000 (2). A variety of factors, such as chronic smoking, high levels of alcohol intake, and human papillomavirus (HPV) or Epstein-Barr virus (EBV) infections, have been indicated to promote HNSC development (3). Conventional HNSC treatment comprises a combination of surgical intervention, radiation, and chemical therapies (4). Recently, with the increasing knowledge of tumor microenvironment (TME), immunotherapy has gained increasing attention and demonstrated remarkable efficacy against various cancers. The PD-1/PD-L1 inhibitor pembrolizumab, which has been shown to improve overall HNSC patient survival, specifically of those with increased PD-L1 receptor expression, has been approved as a first-line HNSC treatment (4–6). However, there are still many patients who do not benefit from pembrolizumab treatment (7). Moreover, the five-year overall survival rate for individuals with HNSC continues to be < 50% (8). Therefore, there’s an urgent need to comprehensively investigate the complexities of the TME and discover new targets for therapeutic intervention.

TRIB3 belongs to the pseudokinase protein family. Despite containing a protein kinase-like domain, key amino acid mutations within this domain result in the loss of its kinase function (9, 10). TRIB3 is well-known for its activity against various “environment stresses,” including insulin-like growth factor-1 (IGF-1), inflammatory factors, and endoplasmic reticulum stress (1). Therefore, it is considered a critical “stress-regulating switch” linking homeostasis, metabolic diseases, and cancer (11). TRIB3 has been reported to be overexpressed in many different malignant tumors, such as clear cell renal cell carcinoma (12), lung cancer (13), breast cancer (14), and gastric cancer (15). This overexpression is often correlated with unfavorable patient outcomes. Furthermore, TRIB3 has been indicated to promote various tumor malignant phenotypes, such as tumor cell proliferation, invasion, and epithelial-mesenchymal transition (13, 16, 17). Recently, the research focus has been shifted towards the role of TRIB3 in modulating immune cells. Wu et al. (12) revealed that in clear-cell renal cell carcinoma, increased TRIB3 expression is negatively correlated with CD8+ T cells and positively correlated with M1 macrophages. In colon cancer, TRIB3 reduces immune infiltration of CD8+ T cells by inhibiting the STAT1-CXCL10 pathway, thereby mediating tumor cell immune evasion (18). Additionally, TRIB3 also modulates immune response in ovarian cancer (19). These data suggest that TRIB3 is an essential molecule affecting tumor prognosis and is closely associated with tumor immune regulation. However, its role in HNSC remains unclear. Therefore, this investigation aims to elucidate the impact of TRIB3 on HNSC prognosis via bioinformatics analysis, evaluate its regulation of the immune microenvironment of HNSC, and validate its effect on the malignant phenotype of HNSCC through *in-vitro* experiments.

## 2 Materials and methods

### 2.1 Differential expression and localization of TRIB3 in cancers

The expression of TRIB3 in 21 different tumors was assessed using the TIMER 2.0 (RRID : SCR_018737) database (20). Additionally, publicly available HNSC-related RNA-sequence data and associated clinical information were acquired from The Cancer Genome Atlas (TCGA, RRID : SCR_003193) database. For the analysis of TRIB3 expression disparities between tumor tissues and their adjacent non-tumor tissues, the “LIMMA (RRID : SCR_010943)” package in R was employed. Lastly, the data was visualized via the “ggplot2 (RRID : SCR_014601)” and “ggpubr (RRID : SCR_021139)” R packages.

To further explore the intracellular expression level and subcellular localization of TRIB3, immunohistochemical staining data of normal and oral squamous cell carcinoma tissues was downloaded from the Human Protein Atlas (HPA, RRID : SCR_006710) database (https://www.proteinatlas.org/).

### 2.2 Sigel-cell analysis of TRIB3 in HNSC

The single-cell analysis of TRIB3 was performed on the Tumor Immune Single-Cell Hub (Tisch) website (21) (http://tisch.comp-genomics.org/) with the following conditions: Gene: TRIB3; cell type annotation: major lineage; all lineage. The data of TRIB3 expression in 16 single-cell types is depicted in a heatmap. Furthermore, the TRIB3 expression level in each cell cluster in the HNSC_GSE103322 and NPC_GSE150430 datasets was analyzed.

### 2.3 Survival analyze

The clinical HNSC patient’s data was obtained from the TCGA database, and Kaplan-Meier (K-M) log-rank tests were employed to assess the influence of TRIB3 on both overall survival (OS) and progression-free survival (PFS) among these patients. The K-M analyses were conducted via the “survival (RRID : SCR_021137)” and “survminer (RRID : SCR_021094)” R packages after categorizing patients into high and low TRIB3 expression cohorts based on a 50% cut-off criterion. Additionally, the ability of TRIB3 to predict the outcomes of HNSC patients was evaluated by establishing the receiver operating characteristic (ROC) curves utilizing the “timeROC” package.

### 2.4 Relationship between TRIB3 and clinical data

To further investigate the potential factors underlying the impact of TRIB3 on prognosis, univariate and multivariate logistic regression analyses were carried out. These were aimed at evaluating the relationships between TRIB3 and various clinical parameters, including age, gender, tumor grade, and TNM and tumor stages. The data were visualized via box plots and heatmaps generated using the “ggplot2” and “ ComplexHeatmap (RRID : SCR_017270) “ packages, respectively.

### 2.5 Prognostic analysis of TRIB3 in HNSC

The univariate and multivariate Cox regression analyses were performed to assess the potential of TRIB3 as an independent prognostic indicator for the outcome of HNSC patients. First, TRIB3, age, gender, tumor grade, and tumor stage were added in the univariate Cox analysis. Since TNM staging had collinearity with tumor stage and TRIB3, it was not included. Subsequently, variables that yielded a p-value <0.05 were subjected to multivariate Cox analysis. Variables with P<0.05 were deemed independent prognostic indicators for HNSC. For the visual representation of these analyses, the “survival” R package was utilized to generate forest plots, which displayed P-values, hazard ratios (HRs), and their respective 95% confidence intervals (CIs). Lastly, a nomogram with all the independent risk factors was established to conveniently predict the 1-, 3-, and 5-year survival prospects of HNSC patients. Calibration curves were plotted to assess the nomogram’s predictive accuracy for these respective time points.

### 2.6 TRIB3 protein-protein interaction, differential expression genes between low- and high-TRIB3 expression groups, and gene fusion analysis

To explore the PPI relationships of TRIB3 in HNSC, PPI genes related to TRIB3 with Pearson coefficients >0.9 were downloaded from the STRING (RRID : SCR_005223) database (22) (https://string-db.org/). Subsequently, correlation analysis was performed on HNSC data from TCGA, and Pearson coefficients were calculated. Genes with Pearson coefficients >0.3 and p-values < 0.001 were selected, and the circos plot was drawn using the “ggplot2” R package a to visualize the top ten positively and negatively co-expressed genes with TRIB3.

Based on the median expression level of TRIB3, HNSC patients were categorized into high- and low-expression cohorts. To identify DEGs between these two subsets, the “LIMMA” R package was employed (23). For the identification of significant differences, the following criteria were set: Log Fold Change (FC) > 0.5 and an adjusted P-value <0.05. The top 50 most significantly upregulated and downregulated genes were visualized via the heatmaps using the “pheatmap (RRID : SCR_016418)” package. Furthermore, Gene Oncology (Gene Ontology, RRID : SCR_002811) and Kyoto Encyclopedia of Genes and Genomes (KEGG, RRID : SCR_012773) analyses were performed (24) on the DEGs using the “clusterProfiler (RRID : SCR_016884)” and “org.Hs.eg.db” packages (25). Additionally, the “c5.all.v2023.1.Hs.symbols” gene set dataset was retrieved from the Gene Set Enrichment Analysis (GSEA, RRID : SCR_003199) website (http://www.gsea-msigdb.org/gsea/index.jsp) to compute normalized enrichment scores (NES) and false discovery rates (FDR) for DEGs between high and low TRIB3 expression cohorts. GSEA-GO analysis (26) was carried out using the “clusterProfiler” package, and the top five most significantly enriched pathways were visualized using the “enrichplot” R package.

### 2.7 Immunotherapy, TME, tumor mutation burden, and checkpoint gene analyses

Using the “estimate” R package (27), the content of tumor cells and immune cells in the TME between high and low TRIB3 expression cohorts was compared. The results were visualized via the violin plots drawn using “ggplot.” Furthermore, the differences in 24 immune cell types in the tumor tissues of the two cohorts were analyzed using the ImmuCellAI_new_method from the “ImmuCellAI” R package (28). Utilizing gene expression data, ImmuCellAI calculated an infiltration score for each type of immune cell for each sample, enabling the estimation of the abundance of 24 distinct immune cell types in the tumor tissue. Following this, the correlation between the quantity of immune cells in the samples and TRIB3 expression was determined using the Spearman method. Correlations yielding a P-value of less than 0.05 were considered statistically significant. “ggplot” was used to draw box and scatter plots to visualize the results. Subsequently, a correlation analysis of TRIB3 with immune checkpoint (ICP) genes was performed, considering P < 0.001 as significant, and the “corrplot” package was employed to visualize the results. Moreover, the impact of TRIB3 on TMB was assessed via pan-cancer TMB files downloaded from the UCSC XENA (29) database (http://xena.ucsc.edu/), and the results were displayed using scatter plots. Lastly, the immunophenoscore (IPS) files of HNSC patients were downloaded from the TCIA (30, 31) database (https://www.tcia.at/home) to predict whether there were any differences in the therapeutic effects of PD1 and CTLA inhibitors between high- and low-TRIB3 expression groups.

### 2.8 Drug sensitivity

To determine whether the expression of TRIB3 could predict the sensitivity of HNSC patients to different drugs, drug sensitivity analysis was performed (32). Half maximal inhibitory concentration (IC50) was used to evaluate the therapeutic response of patients with high- and low-TRIB3 expression to candidate drugs. All the analyses and result visualizations were conducted using “pRRophetic,” “limma,” “ggpubr,” and “ggplot2” packages.

### 2.9 HNSC sample collection

In 2021, a total of 13 pairs of tumor and normal tissue specimens from HNSC patients who underwent surgical resection at the First Affiliated Hospital of Nanchang University were collected. Immediately after surgical resection, ten pairs of tumor and adjacent non-tumor tissue specimens were preserved with RNA preservation solution and stored in a -80°C freezer. The remaining three pairs of tumor and normal squamous epithelial tissue specimens were fixed in formalin for immunohistochemical staining. All samples were collected after informing the patients before the surgery and acquiring their signed consent. The collection of all clinical samples underwent an ethical review and received approval from the Medical Research Ethics Committee of the First Affiliated Hospital of Nanchang University (Ethics number: CDYFYYLK05-013).

### 2.10 Western blot, real-time quantitative polymerase chain reaction and immunohistochemistry

For western blot, total cellular proteins were extracted utilizing RIPA buffers (R0010, Solarbio). The total protein content was then quantified using a BCA protein assay kit (PC0020, Solarbio). Proteins of equal mass were subsequently subjected to SDS-PAGE electrophoresis and transferred onto a PVDF membrane. This membrane was incubated with the primary antibody at 4°C overnight. Finally, the membrane underwent incubation with secondary antibodies at room temperature for 1.5 hours, followed by protein visualization using an Enhanced chemiluminescence kit (S6009M, UElandy).

The RT-qPCR analysis was performed to detect TRIB3 RNA expression differences in 10 pairs of cancer and adjacent non-tumor tissues from HNSC patients. Total cellular RNA was extracted using the RN28-EASYspin Plus Tissue/Cell RNA Rapid Extraction Kit (RN2802, Aidlab) following the manufacturer’s protocol. Subsequently, total RNA (2μg) was reverse-transcribed into cDNA using the SuperScript™ III First-Strand Synthesis System (18080051, Invitrogen). Lastly, for fluorescent quantification, the 2X M5 HiPer real-time PCR mix (MF015m, Mei5bio) was used. The sequences of the primers are detailed in **Supplementary Table 1**.

After resection, the paired samples were immediately fixed with 4% polyformaldehyde for 24 h and then subjected to gradient dehydration, embedding, sectioning, antigen repair, and blocking. Subsequently, the samples were first incubated with an anti-TRIB3 primary antibody at a 1:1000 dilution overnight at 4°C and then for 2 hours with the secondary antibody at room temperature and ultimately counterstained with hematoxylin.

### 2.11 Cell culture and transfection

SCC9 (RRID: CVCL_1685) cell line was purchased from Procell (Wuhan, China), and cultured in a humidified atmosphere with 5% CO2 at 37°C, utilizing DMEM/F12 medium (Gibco, Carlsbad, CA, USA) supplemented with 400 ng/mL hydrocortisone, 10% fetal bovine serum (FBS), and 1% penicillin/streptomycin (P/S). The medium was refreshed every 2-3 days. Cells were passaged upon reaching 70-80% confluence.

The TRIB3 overexpression plasmid was obtained from Zolgene Biotech (Fuzhou, China). A day prior to transfection, SCC9 cells were seeded at 30% density in 6-well plates. The cells underwent transfection with the plasmid and Lipo3000 (L3000015, Invitrogen) for 24 hours. At 48 hours post-transfection, the cells were utilized for subsequent experiments.

### 2.12 Proliferation, migration, and invasion assays

Cell proliferation was assessed using the Cell Counting Kit-8 (CCK-8 kit) (CA1210, Solarbio). SCC9 cells were seeded in 96-well plates at a density of 7 × 10³ cells per well. Subsequently, 110 µl of medium with 10 µl of CCK-8 reagent was added, and the cells were incubated for 2 hours at both 0 and 24 hours. The absorbance at 450 nm was then measured.

The cell migration (without Matrigel) and invasion (with Matrigel) assay was performed using a transwell chamber (3464, corning). Transfected cells, at a concentration of 10 × 10_4_ cells per well, were seeded into the upper chamber with serum-free medium. In the lower chamber, complete medium supplemented with 10% FBS was used. Following a 48-hour incubation period, cells remaining on the membrane of the upper chamber were meticulously removed. The chambers were then fixed with paraformaldehyde and stained with crystal violet. Subsequently, the chambers were photographed and the cells were counted under a microscope.

Cells were seeded in 6-well plates; upon over 90% confluency, a 200-microliter pipette tip was used to create a vertical wound in the center of each well. Images were taken at 0 and 48 hours after the wound was created.

### 2.13 Statistical analysis

To explore the association between TRIB3 and clinical data, both univariate and multivariate logistic regression analyses were utilized. For identifying independent risk factors impacting OS in HNSC patients, univariate and multivariate Cox regression analyses were performed. Survival curves were generated using the KM plotter and comparisons were made through the log-rank test. The Pearson Chi-square test was employed to analyze the correlation between TRIB3 expression and that of other genes. Differences between two groups were evaluated using the t-test. For RT-qPCR analysis of clinical samples, the paired sample t-test was applied. Additionally, one-way ANOVA was used when comparing results across more than two groups. Two-tailed P-value of less than 0.05 was considered indicative of statistical significance. The cell counting in transwell experiments was conducted using IMAGE J software, and graphs were generated using GraphPad PRISM 8.0.

## 3 Results

### 3.1 Widespread genetic alterations of TRIB3 between normal and tumor tissues across different cancer types

**Figure 1:**
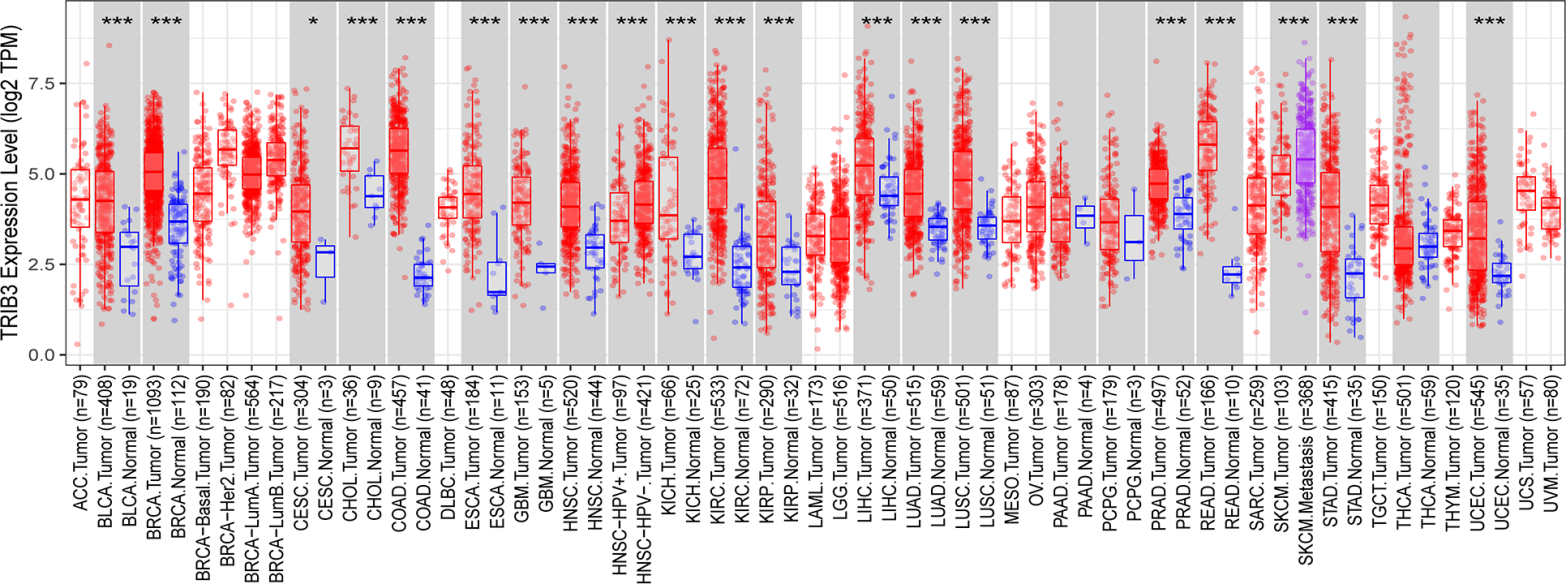
Expression level of TRIB3 in 21 malignant tumors from TIMER2.0 database. (*p value < 0.05; ***p value < 0.001). ACC, Adrenocortical Carcinoma; BLCA, Bladder Urothelial Carcinoma; RCA, Breast Invasive Carcinoma; CESC, Cervical and Endocervical Cancer; CHOL, Cholangiocarcinoma; COAD, Colon Adenocarcinoma; DLBC, Diffuse Large B-cell Lymphoma; ESCA, Esophageal Carcinoma; GBM, Glioblastoma Multiforme; HNSC, Head and Neck Cancer; KICH, Kidney Chromophobe; KIRC, Kidney Renal Clear Cell; Carcinoma; KIRP, Kidney Renal Papillary Cell Carcinoma; LAML, Acute Myeloid Leukemia; LGG, Lower Grade Glioma; LIHC, Liver Hepatocellular Carcinoma; LUAD, Lung Adenocarcinoma; LUSC, Lung Squamous Cell Carcinoma; MESO, Mesothelioma; OV, Ovarian Serous Cystadenocarcinoma; PAAD, Pancreatic Adenocarcinoma; PCPG, Pheochromocytoma and Paraganglioma; PRAD, Prostate Adenocarcinoma; READ, Rectum Adenocarcinoma; SARC, Sarcoma; SKCM, Skin Cutaneous Melanoma; STAD, Stomach Adenocarcinoma; TGCT, Testicular Germ Cell Tumors; THCA, Thyroid Carcinoma; THYM, Thymoma; UCEC, Uterine Corpus; Endometrial Carcinoma; UCS, Uterine Carsinosarcoma; UVM, Uveal Melanoma.

The expression of TRIB3 in 21 malignant tumors was accessed using the TIMER2.0 database. Compared to normal tissues, TRIB3 was significantly upregulated in 17 tumors (BRCA, BLCA, CESC, CHOL, GBM, HNSC, KICH, KIRC, KIRP, LIHC, LUSC, ESCA, PRAD, READ, UCEC, LUAD, and STAD), whereas 4 tumors (LAML, PAAD, PCPG, and THCA) indicated no significant change. TRIB3 expression was markedly higher in SKCM with distant metastasis than in SKCM without distant metastasis (**Figure 1**, **Supplementary Figure 1**).

**Figure 2:**
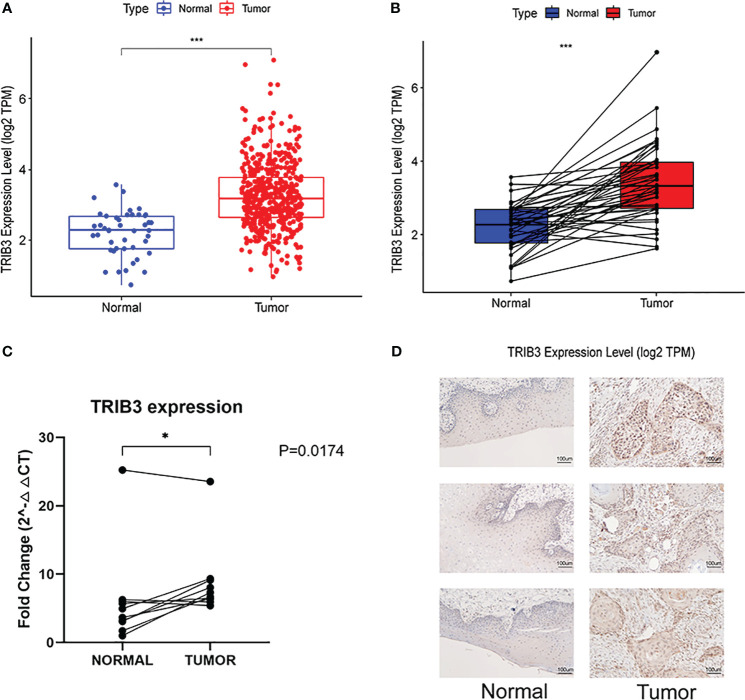
Genetic Alterations of TRIB3 in HNSC. **(A)** Overall TRIB3 Expression level of tumor and normal tissues in HNSC patients from TCGA database. **(B)** Expression of TRIB3 between tumor and paired normal tissues in HNSC patients from TCGA database. **(C)** Real-time qPCR detection of TRIB3 expression in HNSC and paired adjacent normal tissues from clinical samples. Data were analysed by Paired T test. **(D)** IHC images of TRIB3 in HNSC and paired normal squamous epithelial tissues from clinical samples (100×). (*p value < 0.05; ***p value < 0.001). HNSC, Head and Neck squamous cancer; qPCR, quantitative polymerase chain reaction; IHC, Immunohistochemistry.

To further investigate the relationship between TRIB3 and HNSC, RNA-seq data for HNSC patients was acquired from the TCGA database. Then, the expression levels of TRIB3 in overall cancerous and normal tissues (**Figure 2A**), as well as between tumorous tissues and their paired normal counterparts, were compared (**Figure 2B**). Moreover, ten clinical HNSC patients paired samples of tumor and adjacent normal tissues were obtained. As expected, RT-qPCR analyses revealed a significantly elevated expression of TRIB3 in the tumor tissues than in normal adjacent tissues (**Figure 2C**). Immunohistochemical staining further demonstrated that TRIB3 was localized in both the nucleus and the cytoplasm (**Figure 2D**).

### 3.2 TRIB3 is broadly expressed in both malignant tumor cells and immune cells

**Figure 3:**
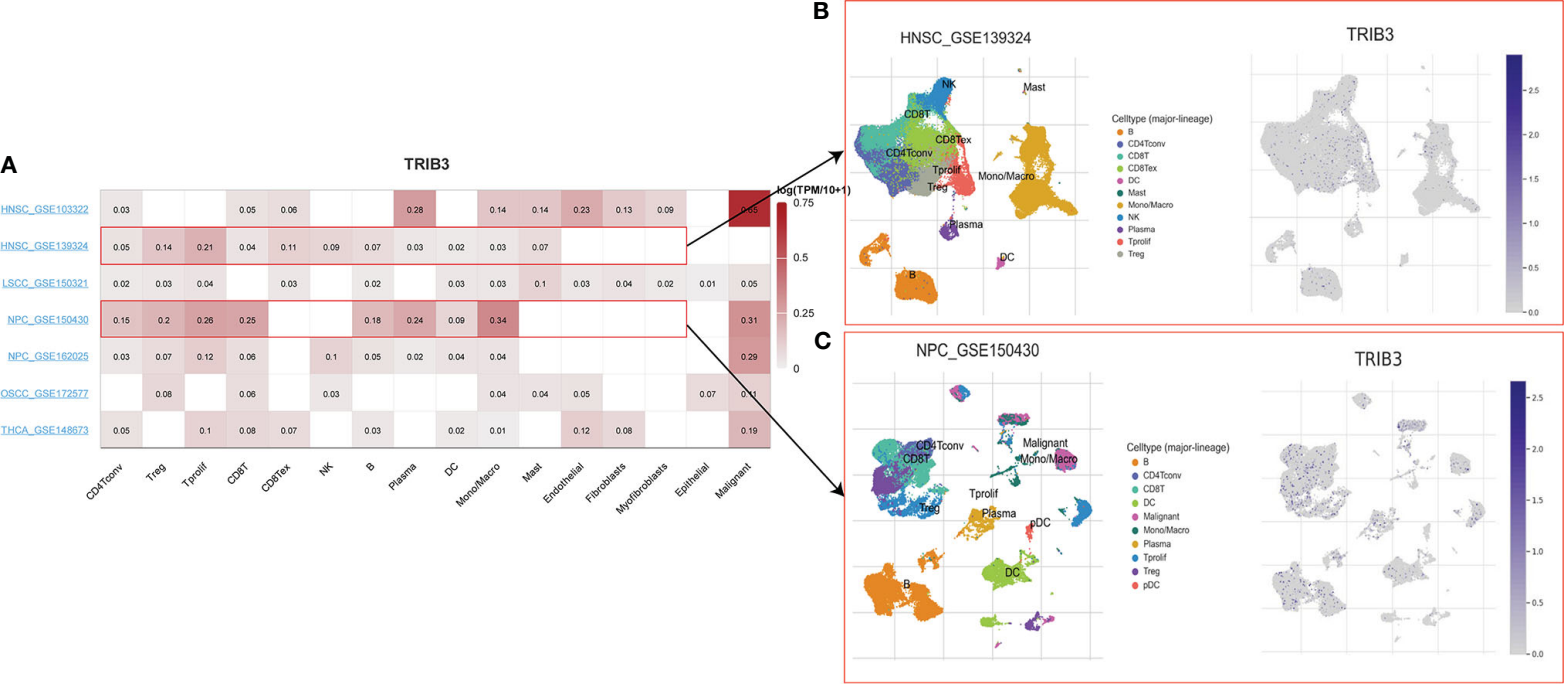
Single-cell sequencing analyses of HNSC. **(A)** Seven datasets of single-cell expression of TRIB3 in HNSC from TISH website; Retrieval strategy: Gene: TRIB3; cell type annotation: major lineage; all lineage. **(B)** The main distributions of TRIB3 on cell types in the HNSC_GSE139324 dataset. **(C)** The main distributions of TRIB3 on cell types in the NPC_GSE150430 dataset. HNSC, Head and Neck squamous cancer; TISH, Immune Single-Cell Hub.

To understand the main cellular distribution of TRIB3 expression in tumor tissues, single-cell sequencing data of 7 HNSC datasets in the TICH database was analyzed. The results showed that TRIB3 was not only expressed in malignant tumor cells but also broadly expressed in most types of immune cells (**Figure 3A**). The HNSC_GSE139324 dataset was used to analyze the gene expression of 130,721 immune cells from 26 patients; TRIB3 was widely expressed in T and natural killer (NK) cells (**Figure 3B**). In the NPC_GSE150430 dataset, 45,959 cells from 15 patients with nasopharyngeal carcinoma were analyzed; TRIB3 shows elevated expression levels in malignant cells as well as in monocytes/macrophages and T cells (**Figure 3C**).

### 3.3 HNSC patients with high TRIB3 expression demonstrated poorer prognosis, which was correlated to the advanced T stage of the tumor

**Figure 4:**
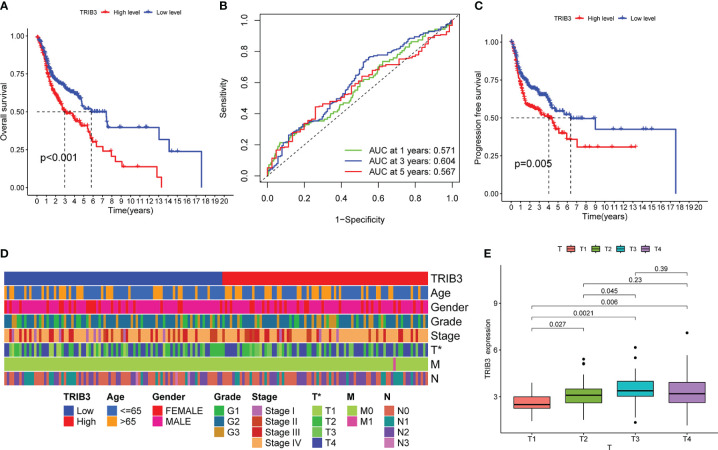
Correlation between TRIB3 expression and HNSC prognosis, as well as clinicopathological data. **(A)** Kaplan–Meier curve showing differences of OS between low- and high-TRIB3 expression HNSC patients. Data were analysed by log-rank test. **(B)** ROC curve, generated by “timeROC” package, showing the ability of TRIB3 in predicting the prognosis of patients at 1-, 3-, and 5-year survival time. **(C)** Kaplan–Meier curve showing differences of PFS between low- and high- TRIB3 expression HNSC patients. **(D)** Correlation Between Clinicopathological Factors and TRIB3 Expression. Data were analysed by univariate and multivariate logistic regression analyze. **(E)** TRIB3 expression in HNSC tissues with different T stages. (*p value < 0.05). HNSC, Head and Neck squamous cancer; OS, Overall survival; ROC, Receiver operating characteristic; PFS, Progression-free survival.

The K-M curve indicated that patients with high TRIB3 expression had a poorer OS rate (**Figure 4A**), whereas the ROC curve revealed that TRIB3 had good accuracy in predicting the prognosis of patients at 1-, 3-, and 5-year survival (**Figure 4B**). PFS is an important indicator of the quality of life of malignant tumor patients; it was revealed that the PFS in the high TRIB3 expression cohort was significantly shorter than low TRIB3 expression cohort (**Figure 4C**), indicating that TRIB3 is an important marker affecting the prognosis and HNSC disease progression, and TRIB3 might be a potential prognostic factor.

To further investigate the relationship between TRIB3 and disease, the relationship between TRIB3 expression and age, gender, grade, and tumor and TNM stages were analyzed (**Figure 4D**). The heatmap displayed the distribution of clinical data for two different expression groups of TRIB3. Furthermore, TRIB3 expression was significantly correlated with the T-stage, and patients with higher T-stage had significantly higher TRIB3 expression than lower T-stage patients (**Figure 4E**), which suggests that TRIB3 expression is related to tumor growth and infiltration ability (33).

### 3.4 TRIB3 serves as an independent prognosis factor for HNSC

**Figure 5:**
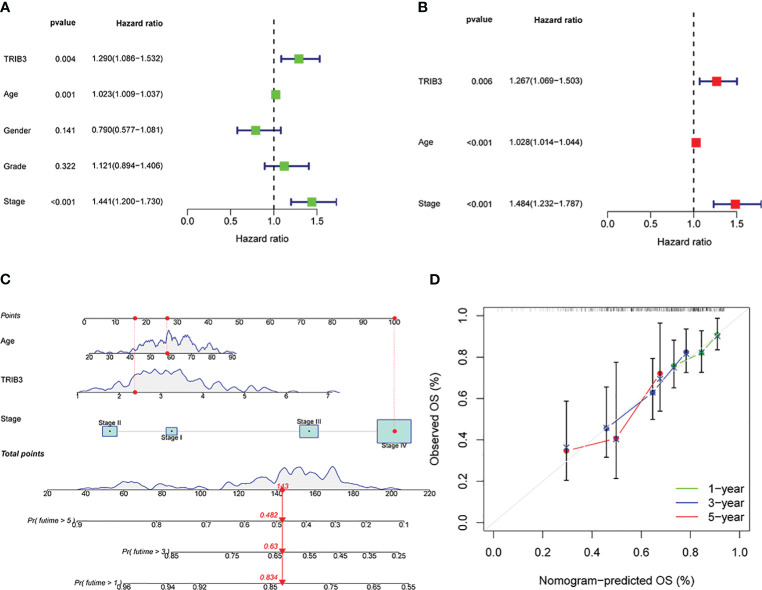
Prognostic significance of TRIB3 in HNSC. **(A)** Univariate Cox regression of TRIB3 in HNSC. Variables with HR values > 1 are risk factors affecting the prognosis of cancer patients, and variables with HR values < 1 are protective factors. **(B)** Multivariate Cox regression of TRIB3 in HNSC. Variables with HR values > 1 are independent prognosis risk factors for HNSC. **(C)** Nomogram model for predicting the OS rates at 1-, 3-, and 5-year of HNSC patients. Points were evaluated by each variable. The points obtained for all variables are then added and marked on the total point axis. To determine the predicted survival probability, a straight line is drawn perpendicularly from this total point mark to intersect with the survival probability axis. **(D)** Calibration curve to evaluate the ability of Nomogram to predict OS at 1-, 3-, and 5-year HNSC patients. HNSC, Head and Neck squamous cancer; HR, Hazard Ratio.

Univariate Cox regression analysis was performed to investigate the impact of age, gender, grade, tumor stage, tumor grade, and TRIB3 expression level on HNSC patient prognosis. Since TRIB3 expression is related to T-stage and TNM is closely related to tumor stage and grading, TNM was not included as a variable in the univariate Cox regression analysis to reduce collinearity between variables. The results showed that age, TRIB3, and tumor stage were potential factors affecting the prognosis of HNSC patients (**Figure 5A**). Subsequently, these potential prognostic factors were included in the multivariate Cox regression analysis, and the results were visualized by forest plot. Multivariate Cox analysis showed that age (HR=1.028, 95% CI = 1.014 to 1.044, p<0.001], TRIB3 expression (HR=1.267, 95% CI = 1.069 to 1.503, p=0.006), and tumor stage (HR=1.484, 95% CI = 1.232 to 1.787, p<0.001) were all independent prognostic factors for HNSC (**Figure 5B**). Based on the above results, a nomogram model was constructed based on these three independent prognostic factors. This model allowed a more intuitive prediction of patient survival probability at 1, 3, and 5 years through the “cumulative scores” of various prognostic factors (**Figure 5C**). Furthermore, the predictive ability of the nomogram model was evaluated. The calibration curve showed that the prediction curve for 1, 3, and 5 years was very close to the ideal curve (**Figure 5D**), indicating that our model has a good predictive ability for HNSC prognosis.

### 3.5 TRIB3 and ER-related genes jointly constitute the PPI network involved in the regulation of HNSC

**Figure 6:**
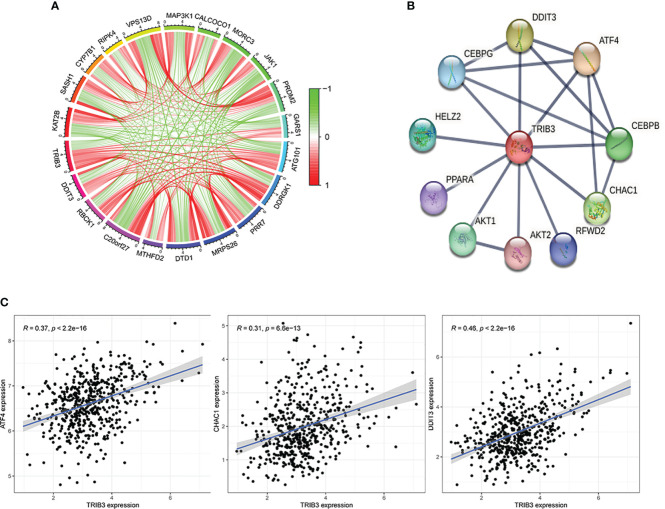
Protein-Protein Interaction of TRIB3 in HNSC. **(A)** The circle plots display the top ten genes that positively and negatively correlated with TRIB3 expression, respectively, in the HNSC-TCGA database according to the Person correlation coefficient. **(B)** Protein-protein interaction network of TRIB3 with the highest confidence (>0.9) from the STRING database. **(C)** Three genes were presented both in the TCGA database with Pearson’s coefficient >0.3 and p<0.001 and in the STRING database with the highest confidence (>0.9). HNSC, Head and Neck squamous cancer.

After the significant prognostic results were indicated for TRIB3 in HNSC, the possible involved biological processes and regulatory pathways were explored. A correlation analysis on HNSC data from the TCGA database was performed, which revealed a total of 138 co-expressed genes (with Pearson’s coefficient >0.3 and p<0.001) with TRIB3. These genes could either directly or indirectly influence TRIB3’s expression, or conversely, be regulated by TRIB3. The circle plots displayed the top ten genes positively or negatively correlated with TRIB3 expression (**Figure 6A**). Furthermore, the PPI network of TRIB3 with the highest confidence (>0.9) from the STRING database was selected (**Figure 6B**). Interestingly, by intersecting the results from these two databases (**Figure 6C**), we discovered that ATF4, DDIT3, and CHAC1 are not only associated with TRIB3 expression in HNSC but also directly interact with the TRIB3 protein. The significance of this finding is that it suggests that these three proteins may play crucial direct regulatory roles in relation to TRIB3 in HNSC patients.

### 3.6 In HNSC, TRIB3 modulates the malignant phenotype of tumor cells and immune response

**Figure 7:**
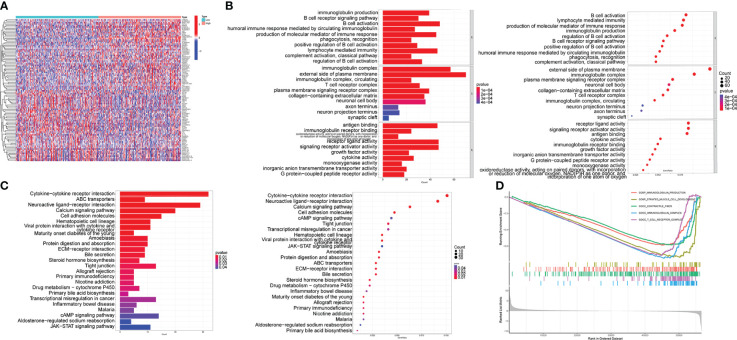
TRIB3 functional enrichment analysis in HNSC. **(A)** Heat maps displaying the top 50 upregulated and top 50 downregulated genes between high- and low-TRIB3 expression groups. Data were analysed by “LIMMA” package. **(B)** Bar-plot and bubble plot depicting enriched pathways obtained from GO analysis of the DEGs. **(C)** GSEA-GO fusion analysis displayed the top 5 significantly enriched regulatory pathways of the DEGs. **(D)** Bar-plot and bubble plot depicting enriched pathways obtained from KEGG analysis of the DEGs. DEGs, Differentially expressed genes; HNSC, Head and Neck squamous cancer.

The DEGs between high- and low-TRIB3 expression cohorts were compared, and 1347 DEGs were obtained, including 636 upregulated and 711 downregulated genes. The heatmaps displayed the top 50 upregulated and downregulated genes, respectively (**Figure 7A**). GO analysis of these DEGs showed that the main differential genes were enriched in many immune function-related pathways, Biological Process (BP): B cell activation, lymphocyte-mediated immunity and regulation of B cell activation; Cellular component (CC): T cell receptor, immunoglobulin, and plasma membrane signaling receptor complexes; Molecular function (MF) enrichments are also immune-related, such as receptor-ligand activity, immunoglobulin receptor binding and cytokine activation (**Figure 7B**). Moreover, GSEA-GO fusion analysis was performed, and the top 5 significantly enriched regulatory pathways were identified. The results showed that immunoglobulin production, immunoglobulin complex, and T cell receptor complex pathways are significantly enriched in the low-TRIB3 expression group (**Figure 7D**), suggesting that fewer immune cells are involved in the TME of high TRIB3 expression HNSC patients. Additionally, KEGG analysis revealed that the main enriched pathways were cell adhesion molecules, tight junction, ECM-receptor interaction, and cytokine-cytokine receptor interaction (**Figure 7C**). These pathways suggest the potential association of TRIB3 with the migratory capability of HNSC cells.

### 3.7 TRIB3 significantly reduced the immune cell infiltration in HNSC TME

**Figure 8:**
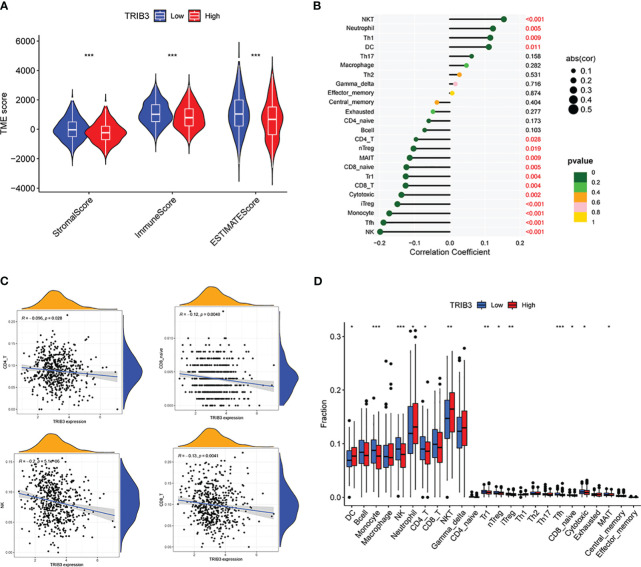
TRIB3 influences the immune cell infiltration in HNSC. **(A)** Violin plots showing the different content of tumor cells and immune cells in the TME between low- and high-TRIB3 expression HNSC patients. **(B)** Correlation between TRIB3 expression and the relative infiltration of 24 immune cell types in HNSC. The color of the dots corresponds to the P value; the size of the dots corresponds to the Spearman correlation coefficient. **(C)** Scatter plots showing the correlation between TRIB3 expression and CD4 Tt, CD8 T, NK, and CD8 naive cell infiltration in HNSC. **(D)** Different infiltration of 24 immune cells between low- and high-TRIB3 expression HNSC patients. (*p value < 0.05; **p value < 0.01; ***p value < 0.001). HNSC, Head and Neck squamous cancer; NK, Natural Killer cells.

As violin plots represent, tumors in high TRIB3 expression patients had fewer stromal and immune cells than those with low TRIB3 expression, i.e., tumors in patients with high TRIB3 expression had a higher proportion of malignant tumor cells (**Figure 8A**). Furthermore, the correlation analysis between TRIB3 and immune cell infiltration, as well as TRIB3 and 24 types of immune cells, was carried out. The results showed that TRIB3 expression was positively correlated with reduced infiltration of NK, Tfh, monocyte, iTreg, Cytotoxic, CD8 T, Tr1, MAIT, nTreg, and CD4 T cells, and increased infiltration of NKT, Neutrophil, Th1, and DC cells (**Figures 8B**, **C**). Upon comparing the immune cell infiltration differences between the high and low TRIB3 expression cohorts, NK, CD4 T, and CD8 T cells indicated a significantly reduced infiltration in the high TRIB3 expression group compared to the low expression group (**Figure 8D**).

### 3.8 TRIB3 isn’t a predictive marker for PD1 and CTLA-4 inhibitor treatments, but it mediates immune evasion in HNSC

**Figure 9:**
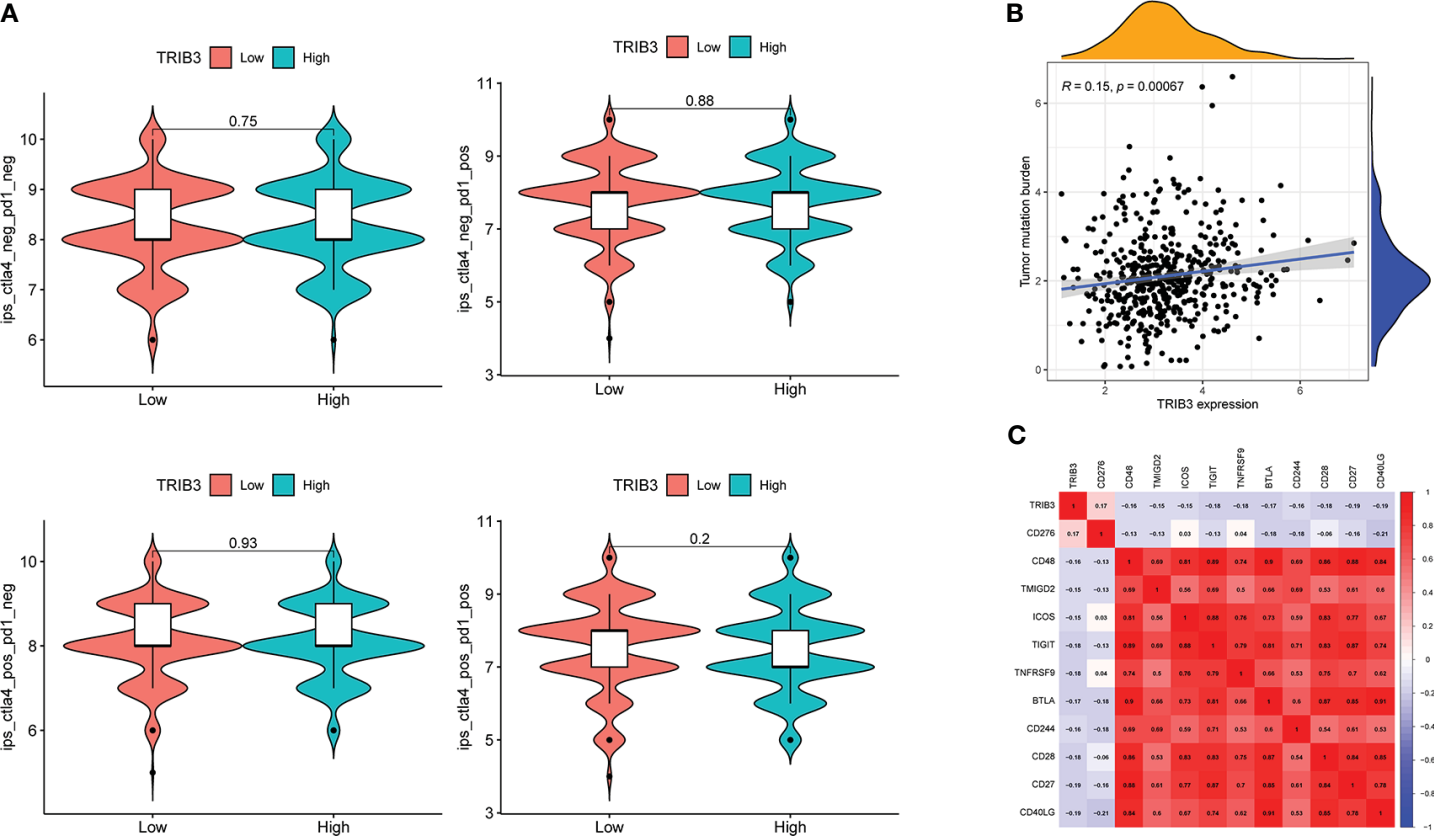
Correlation of TRIB3 expression with immune response in HNSC. **(A)** Violin plot showing whether there is a difference in treatment response to PD-1 and/or CTLA_4 inhibitors in groups with low- and high-TRIB3 expression HNSC patients, respectively. IPS files of HNSC patients were downloaded from TCIA database. **(B)** Scatter plot showing the correlation between TRIB3 expression and TMB in HNSC. TMB file were downloaded from UCSC XENA database. **(C)** Correlation between TRIB3 expression and ICP in HNSC; two-tailed P<0.001 were considered as significant. HNSC, Head and Neck squamous cancer; IPS, immunophenoscore; TMB, Tumor Mutation Burden; ICP, immune checkpoint.

No significant differences in IPS were observed in PD1, ALT4, and PD1+ALT4 between the two groups (**Figure 9A**)., indicating that patients with high TRIB3 expression cannot benefit from PD1 and ALT4 ICP inhibitor treatments. However, the TMB analysis indicated that the high TRIB3 expression group was significantly higher than the low expression group (**Figure 9B**), implying that high TRIB3 expression patients have better immune responses (34). Therefore, to explore potential immune targets, a correlation analysis between TRIB3 and 47 well-known ICP genes was carried out (12). The results showed that despite being positively correlated with CD276, TRIB3 was significantly negatively correlated with CD48, TMIGD2, ICOS, TIGIT, TNFRSF9, BTLA, CD244, CD28, CD27, and CD40LG (**Figure 9C**).

### 3.9 TRIB3 affects the sensitivity of HNSC patients to specific drugs

**Figure 10:**
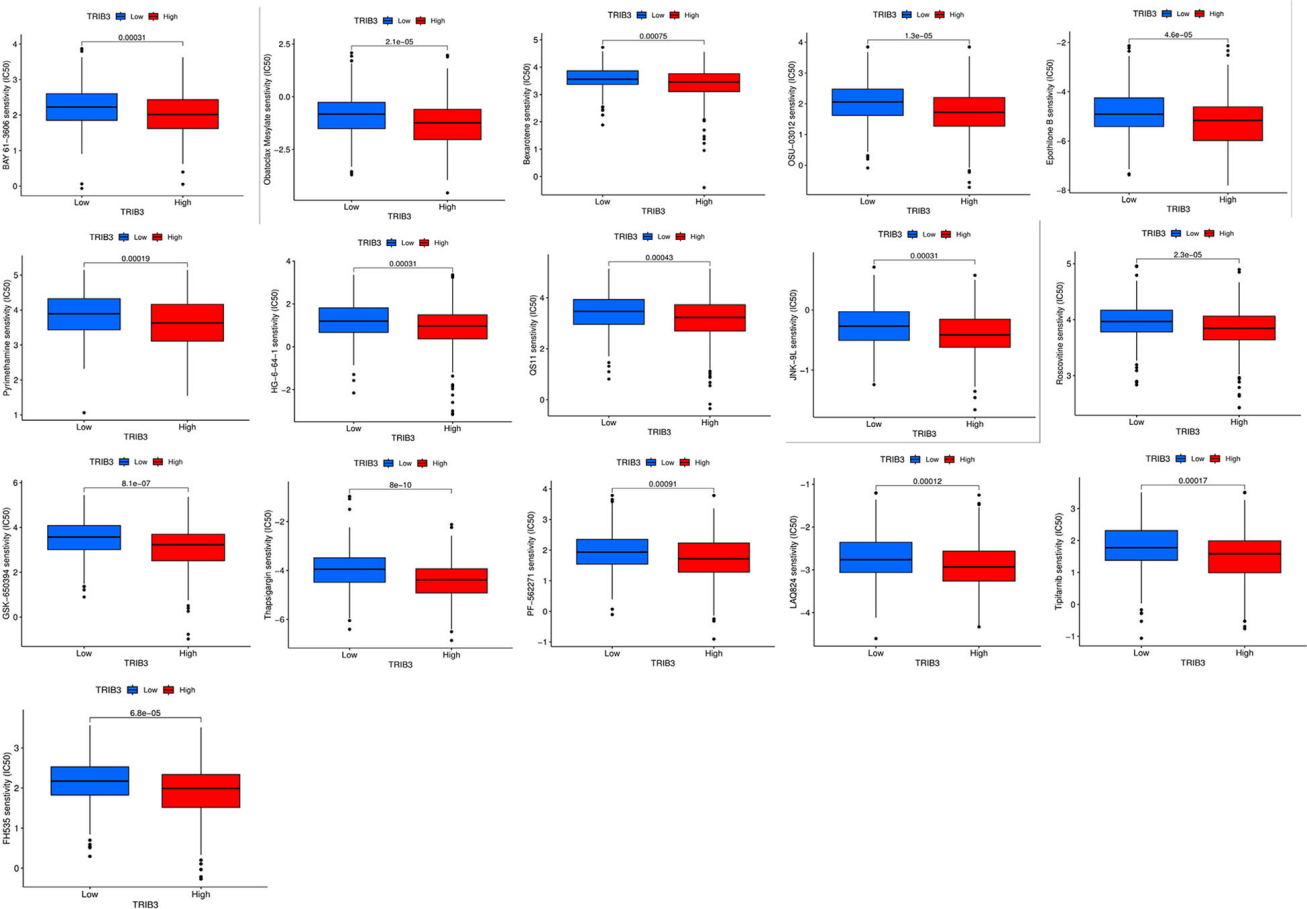
Candidate potential sensitive drugs for low- and high-TRIB3 expression HNSC patients. Patients with high TRIB3 expression had lower IC50 values for Obatoclax mesylate, Bexarotene, Osu-03012, BAY61-3606, Epothilone, Pyrimethamine, Roscovitine, LAQ824, PF-562271, FH535, GSK-650394, HG-6-64-1, QS11, JNK-9L, Tipifarnib, Thapsigargin.

**Figure 11:**
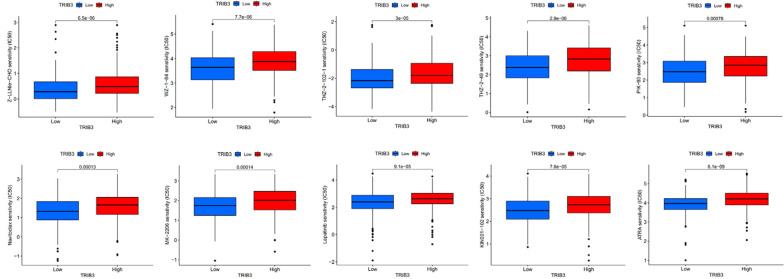
Patients with low TRIB3 expression had lower IC50 values for Z-LLNIe-CHO, WZ-1-84, THZ-2-102-1, THZ-2-49, PIK-93, Naritoclax, MK-2206, Lapatinib, KIN001-102, ATRA.

Increased TRIB3 expression patients indicated reduced IC50 values of Obatoclax mesylate, Bexarotene, Osu-03012, BAY61-3606, Epothilone, Pyrimethamine, Roscovitine, LAQ824, PF-562271, FH535, GSK-650394, HG-6-64-1, QS11, JNK-9L, Tipifarnib, Thapsigargin, indicating that these patients have better sensitivity towards these drugs (**Figure 10**). Whereas patients with low TRIB3 expression had lower IC50 values of Z-LLNIe-CHO, WZ-1-84, THZ-2-102-1, THZ-2-49, PIK-93, Naritoclax, MK-2206, Lapatinib, KIN001-102, ATRA, suggesting that they were more sensitive to these drugs (**Figure 11**).

### 3.10 *In vitro* validation of TRIB3 gene function

**Figure 12:**
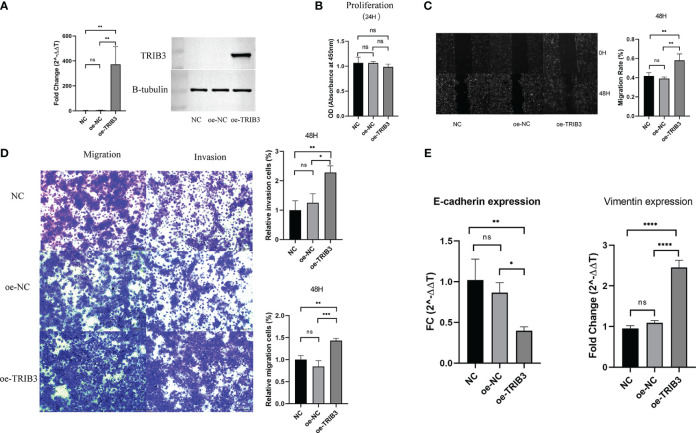
*In vitro* validation experiment. **(A)** Validation experiments to verify TRIB3 overexpression in SCC9 cells by RT-qPCR and Western Blot. Data were analysed by multiple comparisons of one-way ANOVA analyse. **(B)** Overexpression of TRIB3 did not change the proliferation ability of SCC9 cells. Data were analysed by multiple comparisons of one-way ANOVA analyse. **(C)**Wound-healing assay showing SCC9 with overexpressing TRIB3 had a significantly higher migration rate than the NC and oe-NC groups at 48 hours. Data were analysed by multiple comparisons of one-way ANOVA analyse. **(D)** Transwell assays (with and without Matrigel) revealed that the overexpression of TRIB3 markedly enhances the migration (left) and invasive (right) capabilities of SCC9 cells. Data were analysed by multiple comparisons of one-way ANOVA analyse. **(E)** RT-qPCR analysis demonstrates that overexpression of TRIB3 facilitates the EMT process, indicated by a reduction in E-cadherin expression and an upgradation in Vimentin expression. Data were analysed by multiple comparisons of one-way ANOVA analyse. (*p value < 0.05; **p value < 0.01; ***p value < 0.001; ****p value < 0.0001). ns, no significance; EMT, epithelial-mesenchymal transition; RT-qPCR, Reverse Transcription Quantitative Polymerase Chain Reaction; NC, Control; oe-NC, Negative Control for transfection; oe-TRIB3, TRIB3 overexpression.

Initially, the transfection efficacy in SCC9 cells was validated using RT-qPCR (**Figure 12A**) and Western blot analysis (**Figure 12B**). Subsequent assessment using the CCK8 assay revealed that the overexpression of the TRIB3 gene did not significantly impact the proliferation capacity of SCC9 cells. Following this, a migration assay was conducted, showing that 48 hours post-transfection, SCC9 cells with overexpressed TRIB3 exhibited a notably higher migration rate compared to both the control and negative control groups (**Figure 12C**). Furthermore, the Transwell experiment corroborated that TRIB3 substantially enhances the migration and invasion abilities of SCC9 cells (**Figure 12D**).

In a concluding set of experiments focusing on the key aspects of cell adhesion, we performed qPCR validation for EMT markers, namely vimentin and E-cadherin. The results demonstrated that TRIB3 overexpression facilitates the EMT process, as evidenced by a decrease in E-cadherin expression and an increase in vimentin expression, signifying a shift towards a mesenchymal phenotype (**Figure 12E**).

## 4 Discussion

Pembrolizumab has achieved significant breakthroughs in immunotherapy for HNSC treatment (4). However, the variability in the tumor-suppressive microenvironment among different patients suggests that pembrolizumab is only effective for a few HNSC patients (7). Therefore, a search for new potential biomarkers and immune targets is urgently needed.

TRIB3 protein belongs to the pseudokinase family and is closely associated with the onset of various cancers. Stress-induced TRIB3 expression enhances the resistance of cancer cells to hypoxia and is associated with poor prognosis in breast cancer (14). Furthermore, it has been indicated that TRIB3 has a pro-tumorigenic function in renal clear cell carcinoma, liver cancer, lung adenocarcinoma, and acute promyelocytic leukemia (12, 13, 35, 36). Recently, much research has found that TRIB3 plays a crucial role in tumor immune regulation (12, 18, 19). However, a comprehensive understanding of its function in the progression of HNSC remains undetermined. In this study, it was discovered that TRIB3 is a potent prognostic biomarker for HNSC, closely associated with immune evasion in solid tumors and immune cell infiltration. These findings can provide new insights for further investigation into the potential role of TRIB3 in HNSC.

Here, the expression status of TRIB3 transcriptomic data across 21 tumor types was assessed, and it revealed that TRIB3 RNA levels were elevated in almost all tumors. Furthermore, its expression in HNSC in paired and unpaired samples indicated significant upregulation compared to normal tissues. To corroborate this, the clinical HNSC samples were subjected to the qPCR, which confirmed the elevated expression of TRIB3 in HNSC-paired samples. As proteins serve as the final functional units in biology, immunohistochemical analysis was performed; the results were consistent with the findings of Zhang et al. (37). TRIB3 expression is notably elevated in HNSC cells compared to normal tongue squamous epithelium, and it is expressed in both the nucleus and cytoplasm. The immunohistochemistry results for oral squamous cell carcinoma in the HPA database further corroborate the reliability of the acquired data.

Solid tumors are comprised of malignant, various immune, and stromal cells (38). To deeply understand the function of TRIB3 in solid tumors, it’s crucial to identify its location and distribution. Single-cell analysis indicates that TRIB3 is primarily expressed in malignant cells and is also broadly expressed in various types of immune cells. Notably, TRIB3 is expressed in CD4T/CD8T cells and monocyte-macrophages across almost all datasets, consistent with the findings from single-cell data on renal cancer (12). Moreover, TRIB3 has been reported to affect the immune infiltration of NK, T, and B cells in colorectal cancer (39). This suggests, to some extent, that TRIB3 participates in the progression of HNSC, not just through malignant cells. Therefore, immune cells within solid tumors should also be studied.

Additionally, the prognostic significance of TRIB3 for HNSC patients and its clinicopathological relevance were assessed. The survival curves indicated that elevated expression of TRIB3 is associated with a lower OS rate and disease-free progression. Clinical data analysis revealed that the TRIB3 expression level is significantly correlated with the T-stage of HNSC patients, with patients at advanced T-stages showing progressively increased TRIB3 expression. According to the independent prognostic analysis, age, grade, and TRIB3 jointly serve as independent prognostic factors for HNSC patients, consistent with previous discoveries in lung cancer, breast cancer, and liver cancer, all suggesting that TRIB3 acts as an oncogene involved in the progression of malignant tumors, causing poorer prognosis (13, 14, 35). Subsequently, the constructed nomogram model could precisely predict the impact of these three risk factors on the survival rate of HNSC patients.

This research also investigated genes that are significantly associated with TRIB3 expression in HNSC, which revealed that these genes are also abnormally expressed and directly or indirectly form a regulatory network with TRIB3 that impacts the onset and progression of HNSC. Subsequently, the genes interacting with TRIB3 protein were retrieved from the STRING database, and those that directly interacted with TRIB3 were filtered out. Three key genes (ATF4, DDIT3, and CHAC1) were identified. ATF4 is an endoplasmic reticulum (ER) stress-related gene that plays a role in activating TRIB3 expression (40). Additionally, ATF4 is a crucial transcription factor for activating NRF2 transcription, which ensures cell survival under adverse conditions (41). DDIT3 is a vital downstream ER stress gene that regulates autophagy (42). CHAC1’s expression also increases during ER and oxidative stress (43) and is viewed as a risk prognostic factor for breast cancer and renal clear cell carcinoma (44, 45). These findings suggest that the activation of TRIB3 might be closely associated with the ER stress response, and these genes collectively endow tumor cells with the ability to resist harsh environments.

To better understand the function of the TRIB3 and its associated activation pathways, a gene set enrichment analysis was carried out. KEGG analysis indicated that TRIB3 is involved in the malignant phenotype regulation of HNSC. Furthermore, it participates in the cell adhesion molecules, tight junction, and ECM-receptor interaction pathways, which are associated with the migration and invasion function of tumor cells (46). GO and GSEA analyses indicated that TRIB3 extensively regulates immune-related pathways. These findings suggest that TRIB3, as an oncogene, regulates a tumor’s immune microenvironment.

Based on these crucial findings, the content of immune cells in the HNSC TME was assessed. TRIB3 significantly influences the infiltration of immune cells within solid tumors. NK, CD4 T, and CD8 T were considerably reduced in the high TRIB3 expression cohort compared to the low expression cohort, in line with our single-cell analysis, both methods emphasize the vital role of CD4 T and CD8+ T cells in the HNSC TME. NK cells are innate immune cells capable of directly recognizing the expression of major histocompatibility complex class I (MHC-I) molecules and eliminating foreign or stressed target cells, such as those affected by viral infections, aging, or cancer transformation (47). These findings suggest that TRIB3 plays a significant regulatory role in solid tumors. Tumor TRIB3 is primarily located in malignant cells, mediating their progression, and it is also widely expressed in the immune cells of the TME. High TRIB3 expression inhibits immune cell infiltration, typically associated with a weaker immune response. This also explains the association of higher TRIB3 expression with poorer prognosis in patients.

Cancer cells evade immune surveillance by expressing ICP antigens. PD-1/PD-L1 and CTLA-4 are well-known immunosuppressive ICP genes, and their inhibitors are now widely used clinically (48). However, the efficacy of these inhibitors in specific individuals remains uncertain. Given the strong correlation between TRIB3 expression and immune regulation, the influence of PD-1/PD-L1 and CTLA-4 inhibitors on TRIB3 expression was predicted. The findings indicate that these inhibitor treatments did not lead to better outcomes for patients. Fortunately, some other ICP genes including CD276, CD48, TMIGD2, ICOS, TIGIT, TNFRSF9, BTLA, CD244, CD28, CD27, and CD40LG were highly correlated with TRIB3 expression. Like PD-1 and CTLA-4, CD276 is also an immunosuppressive ICP gene and has been associated with poorer prognosis and advanced HNSC TNM staging (49). Moreover, CD276 is a key molecule causing HNSC cancer stem cells to evade immune surveillance and is closely related to the CD8+T cell-dependent elimination of cancer stem cells (50). CD244 is a high-affinity ligand for CD48. CD276 and CD244 have been reported as crucial for the activation of effector T and NK cells (51). TNFRSF9 (also called CD137) agonists have recently been found to effectively activate T cells in combination with PD1 inhibitors and are associated with the recruitment and intratumoral expansion of CD8+T cells (52). The association of TRIB3 with these ICPs further solidifies its potential role in mediating immune evasion in HNSC. In the future, we anticipate more evidence elucidating the association between TRIB3 and ICPs. It is hoped that subsequent studies can provide detailed insights into the specific mechanisms that underpin the regulation between TRIB3 and ICPs.

Beyond immunotherapies, drug treatment remains a primary therapeutic strategy for HNSC. This investigation predicts the chemotherapeutic agents that might demonstrate heightened sensitivity in patients to offer optimal potential regimens for HNSC patients. However, it is important to note that most of these drugs are not standard treatment for HNSC, and our prediction aims to inform how tumor samples with distinct characteristics might react to various chemotherapeutic agents, thus aiding in the customization of treatment plans. However, it’s critical to acknowledge that the precision of these predictions can be influenced by several factors, such as the representativeness of the drug screening data and inherent limitations of the predictive model. Consequently, while this method offers significant insights, it should be prohibited from clinical use until proven.

Although this research has established TRIB3 as a promising therapeutic target for HNSC, which participates in the regulation of the immune microenvironment and is closely associated with prognosis, there are a few limitations. Firstly, the specific mechanism by which TRIB3 modulates immune cells within the TME requires validation through a series of meticulously designed experiments. Additionally, the application of TRIB3 as a prognostic HNSC marker needs confirmation in bench-to-bedside clinical applications. Nonetheless, this outstanding information highlights the future avenues of research, aiming to shed potential insights into the development of novel treatment modalities for HNSC.

In conclusion, this study explored the role of TRIB3 for prognostic prediction, immune cell infiltration, immune evasion, immune-related markers, and drug sensitivity in HNSC. The acquired data suggest that TRIB3 is a powerful and promising biomarker for predicting HNSC prognosis and is associated with immune evasion and immune cell infiltration in HNSC.

## Data availability statement

The datasets presented in this study can be found in online repositories. The names of the repository/repositories and accession number(s) can be found in the article/**Supplementary Material**.

## Ethics statement

The studies involving humans were approved by Medical Research Ethics Committee of the First Affiliated Hospital of Nanchang University. The studies were conducted in accordance with the local legislation and institutional requirements. The participants provided their written informed consent to participate in this study.

## Author contributions

HDW: Data curation, Formal analysis, Methodology, Software, Visualization, Writing – original draft, Writing – review & editing. ZF: Methodology, Software, Visualization, Writing – original draft, Writing – review & editing. HL: Writing – original draft, Writing – review & editing. FF: Validation, Writing – original draft, Writing – review & editing. BH: Resources, Writing – review & editing. YY: Writing – original draft, Writing – review & editing. HYW: Writing – original draft, Writing – review & editing. DX: Resources, Writing – review & editing. HZ: Validation, Writing – review & editing. QZ: Funding acquisition, Project administration, Supervision, Writing – review & editing.
