# Supplementary figures and images for: TRIB3, as a robust prognostic biomarker for HNSC, is associated with poor immune infiltration and cancer cell immune evasion
Source: Front Immunol. 2024 Jan 3;14:1290839. doi: 10.3389/fimmu.2023.1290839 (PMC10791810; doi:10.3389/fimmu.2023.1290839)

## Slide 1
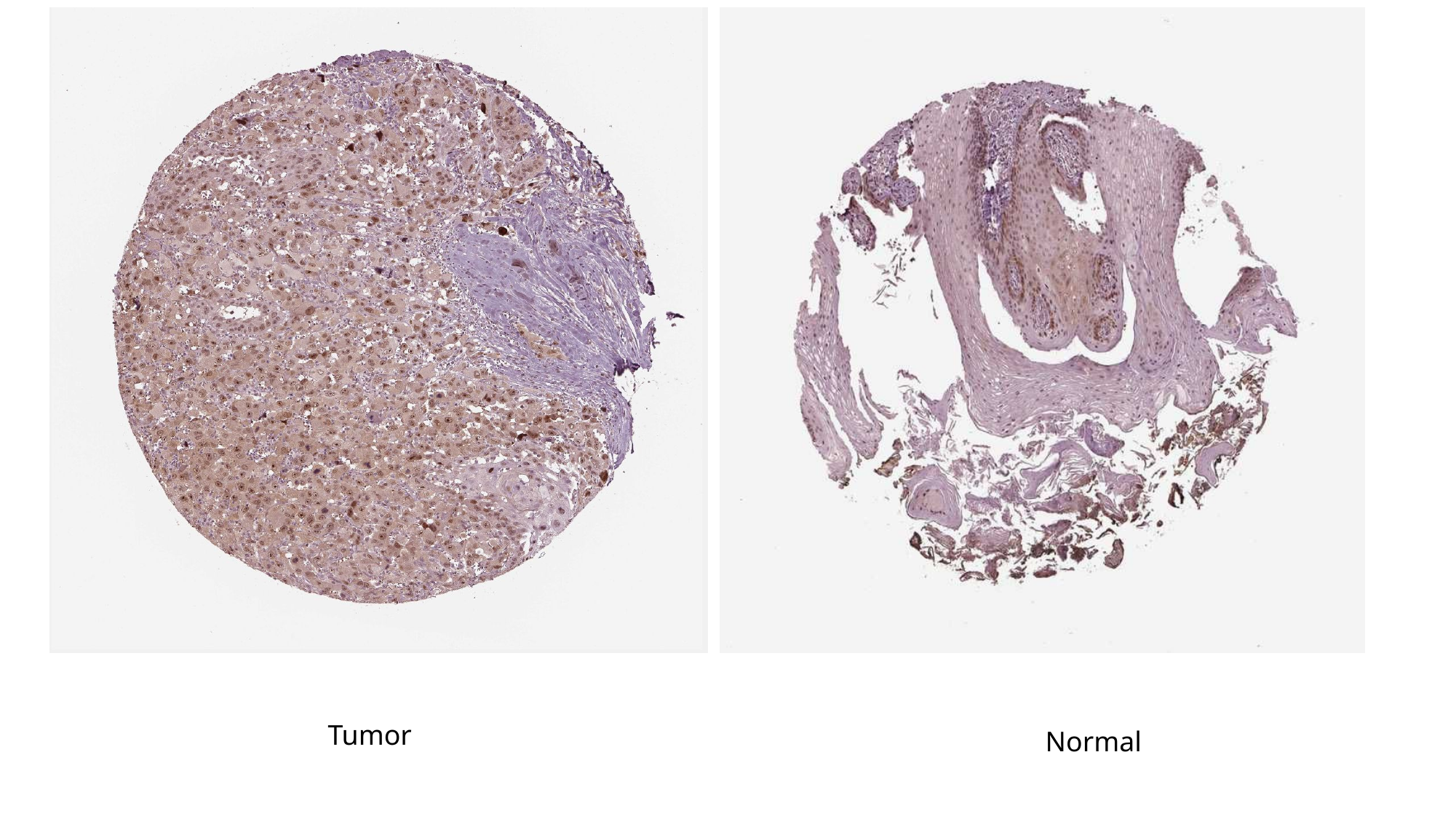

Tumor
Normal

Supplement: Supplementary file 1 [file Presentation_1.pptx]
